# Nephrin and Podocin functions are highly conserved between the zebrafish pronephros and mammalian metanephros

**DOI:** 10.3892/mmr.2013.1844

**Published:** 2013-12-06

**Authors:** YAYOI FUKUYO, TOMOMI NAKAMURA, EKATERINA BUBENSHCHIKOVA, REBECCA POWELL, TAKASHI TSUJI, RALF JANKNECHT, TOMOKO OBARA

**Affiliations:** 1 Department of Cell Biology, University of Oklahoma Health Sciences Center, Oklahoma City, OK 73104, USA; 2 Department of Biological Science and Technology, Graduate School of Industrial Science and Technology, Tokyo University of Science, Noda, Chiba 278-8510, Japan

**Keywords:** human metanephros, nephrin, nephrotic syndrome, podocin, zebrafish pronephros

## Abstract

The slit diaphragm (SD) is a highly specialized intercellular junction between podocyte foot processes and is crucial in the formation of the filtration barrier in the renal glomeruli. Zebrafish Nephrin and Podocin are important in the formation of the podocyte SD and mutations in *NEPHRIN* and *PODOCIN* genes cause human nephrotic syndrome. In the present study, the zebrafish Podocin protein was observed to be predominantly localized in the pronephric glomerular podocytes, as previously reported for Nephrin. To understand the function of Podocin and Nephrin in zebrafish, splice-blocking morpholino antisense oligonucleotides were used. Knockdown of Podocin or Nephrin by this method induced pronephric glomerular hypoplasia with pericardial edema. Human *NEPHRIN* and *PODOCIN* mRNA rescued this glomerular phenotype, however, the efficacy of the rescues was greatly reduced when mRNA-encoding human disease-causing NEPHRIN-R1109X and PODOCIN-R138Q were used. Furthermore, an association between zebrafish Nephrin and Podocin proteins was observed. Notably, Podocin-R150Q, corresponding to human PODOCIN-R138Q, markedly interacted with NEPHRIN compared with wild-type PODOCIN, suggesting that this strong binding capacity of mutated PODOCIN impairs the transport of NEPHRIN and PODOCIN out of the endoplasmic reticulum. The results suggest that the functions of Nephrin and Podocin are highly conserved between the zebrafish pronephros and mammalian metanephros. Accordingly, the zebrafish pronephros may provide a useful tool for analyzing disease-causing gene mutations in human kidney disorders.

## Introduction

The glomerulus is a common structural organization among vertebrates and kidney types (pronephros, mesonephros and metanephros) (1–4). The glomerular filtration barrier, which is responsible for the size and charge-selective properties of the filter, comprises a fenestrated endothelium, composed of the glomerular basement membrane and the podocyte, which are highly specialized epithelial cells of the kidney. The podocyte is composed of three subcellular compartments: a cell body, major processes that extend outward from the cell body and more distally located foot processes that are spanned by a slit diaphragm (SD), which is an essential element of the filtration barrier (5). This basic cellular architecture of podocytes is conserved in vertebrates (6,8).

Intensive research has focused on the molecular basis of SD structure and function and has resulted in the identification of a number of novel SD-associated proteins, including NEPHRIN, NEPH1, CD2-associated protein and PODOCIN (1-5,9-22). These SD-specific proteins are crucial in the formation of the SD and mutations in the *NEPHRIN* and *PODOCIN* genes cause congenital nephrotic syndrome of the Finnish type and autosomal recessive steroid-resistant nephrotic syndrome, respectively (23,25). Numerous disease-associated mutations have been reported in the *NEPHRIN* and *PODOCIN* genes (25,26), however, the information on the functional abnormalities induced by gene mutations in *NEPHRIN* and *PODOCIN* remains limited.

Zebrafish homologues of Nephrin and Podocin are predominantly expressed in the pronephric glomerulus, which is similar to their expression in mammalian metanephric glomerulus and play crucial roles in the formation and function of the SD in the zebrafish pronephric glomerulus (3,9,27). Three morpholino antisense oligos (MOs) used in zebrafish to target Nephrin and Podocin resulted in failure to form normal podocyte architecture, including regular foot processes and SD in zebrafish larvae (9,22). However, updated zebrafish genomic DNA sequences and Gene Tools LLC no longer support these previously used MOs. In the present study, the role of Nephrin and Podocin in the zebrafish pronephros glomerulus was analyzed using different MOs and their evolutionary conservation with human homologues was assessed.

## Materials and methods

### Fish maintenance

The animal experiments were performed in strict accordance with the recommendations in the Guide for the Care and Use of Laboratory Animals of the National Institutes of Health and were approved by the Institutional Animal Care and Use Committee of the University of Oklahoma Health Sciences Center (IACUC protocol no. 12-033 to T.O.). The AB strain of zebrafish was maintained at 28.5°C under a 14 h light/10 h dark cycle. Embryos were maintained at 28.5°C in 0.5X E2 egg medium.

### Cloning of zebrafish nephrin and podocin

Full-length zebrafish *nephrin* cDNA that was subcloned into the pCR-BluntII-TOPO vector was a kind gift from Dr Iain Drummond (9). Full-length zebrafish *podocin* cDNA was obtained by performing RT-PCR on the total RNA isolated from 4 days post-fertilization (dpf) embryos using RNAqueous-4PCR kit (Life Technologies, Carlsbad, CA, USA) and subsequently performing nested PCR. RT-PCR was performed using SuperScript III One-Step RT-PCR System with Platinum Taq High Fidelity (Life Technologies) and nested PCR using Phusion High-Fidelity DNA Polymerase (ThermoScientific, Waltham, MA, USA). The primer sets used were: RT-PCR, forward: 5′-ATC TGC ACT GGC CTC CTG ATA-3′ and reverse: 5′-ATG CGA AGG AAA TCC GTC AAC-3′ and nested PCR, forward: 5′-CAC CAG AGG ACA CTT CAC AAC A-3′ and reverse 5′-CAG CCA ATA ATC AGT ACA GTC TTG AAA-3′. *Podocin* cDNA was subcloned into pCR-BluntII-TOPO and verified by DNA sequencing.

### In situ hybridization

*In situ* hybridization was conducted as previously described (1–3,9). In brief, the pCR-BluntII-TOPO- *nephrin* and -*podocin* digested with *Xho*I were used as a template for anti-sense RNA probe. The probe was synthesized with an SP6 RNA polymerase (New England BioLabs, Ipswich, MA, USA) and DIG-RNA labeling kit (Roche Diagnostics, Mannheim, Germany). Embryos were fixed in 4% paraformaldehyde (PFA), 0.1% Tween-20 in PBS for 2 h at room temperature (RT), altered to 100% methanol and stored at −20°C. Whole-mount *in situ* hybridization was performed as previously described (28). Following color development, the samples were dehydrated with a graded series of methanol and embedded in JB-4 resin (Polysciences, Inc., Warrington, PA, USA). Ten micron sections were sliced using an RN2255 microtome (Leica Microsystems, Wetzlar, Germany) and counter-stained with special eosin II (BBC Biochemical, Mount Vernon, WA, USA). After mounting in Poly-Mount (Polysciences, Warrington, PA, USA), the stained sections were photographed on a Provis AX-70 microscope (Olympus, Tokyo, Japan) equipped with a RETIGA EXi digital camera (QImaging, Surrey, Canada).

### Antibodies

A polyclonal anti-zebrafish Nephrin antibody was prepared as previously described (3,21). Rabbit polyclonal anti-zebrafish Podocin antibody was raised in rabbits using the amino-terminal peptide VKLQEPHKRKE (amino acids 43–53) coupled to KLH. The antiserum was affinity-purified against the immunizing peptide (Covance, Denver, PA, USA).

### Immunoblot analysis and immunohistochemistry

Proteins were extracted from 1 to 5 dpf zebrafish. Prior to protein extraction, the yolk ball was removed from 4 dpf zebrafish larvae, as a large amount of yolk-derived proteins occasionally affects SDS-PAGE and immunoblot analysis (29). Deyolked larvae were homogenized and solubilized in the protein extraction buffer (1% NP-40, 150 mM NaCl, 50 mM KI, 1 mM EDTA and 10% glycerol in 50 mM HEPES, pH 7.4). The homogenate was centrifuged at 11,300 × g for 10 min and the supernatant was collected as the lysate. Each lysate containing 50 μg of protein was subjected to SDS-PAGE (Mini-PROTEAN TGX Gel, 4–15% gradient; Bio-Rad Laboratories, Hercules, CA, USA) and separated proteins were transferred onto a PVDF membrane (Immobilon Transfer Membrane; Millipore, Billerica, MA, USA). Each membrane was incubated with the primary antibody for 2 h at RT and then with an anti-HRP-conjugated secondary antibody (working dilution 1:5,000; Jackson ImmunoResearch Laboratories, West Grove, PA, USA) for 1 h at RT. Dilutions of primary antibodies were 1:2,500 for anti-Nephrin, 1:5,000 for anti-Podocin and 1:5,000 for anti-α-tubulin.

### Immunohistochemical detection of Nephrin and Podocin

Immunohistochemical analysis was performed as previously described (3). Larvae were fixed with Dent’s fixative (20% DMSO in methanol) overnight at 4°C. Fixed samples were rehydrated with a graded series of methanol and washed with PBS containing 0.5% Triton X-100 (PBSTx). For antigen retrieval, the samples were heated in Antigen Retrieval Reagent Universal (R&D Systems, Minneapolis, MN, USA) for 15 min at 95°C on a heat block. Subsequently, the samples were blocked with the incubation solution (PBSTx containing 10% normal goat serum and 1% DMSO) for 2 h at RT and incubated with the primary antibody (working dilution 1:100) in the incubation solution for 12 h at 4°C. After washing with PBSTx, the samples were incubated with Alexa-Fluor546-conjugated goat anti-rabbit IgG (H+L; Jackson ImmunoResearch Laboratories) and diluted with the incubation solution (1:1,000) for 2 h at RT. Stained samples were dehydrated with a graded series of methanol, embedded in JB-4 resin (Polysciences, Inc.) and sliced into 10-μm sections with a RN2255 microtome. The sections were photographed with an FV-1000 confocal laser scanning microscope (Olympus).

### Production of Flag-tagged zebrafish podocin

cDNA corresponding to zebrafish *podocin* was generated using RT-PCR, digested with *Bam*HI and *Xba*I, subcloned into pEV3S-Flag vector and verified by DNA sequencing. This cDNA was used to generate the R150Q mutation, which was also cloned into the pEV3S-Flag vector and verified by DNA sequencing. Human embryonic kidney 293T cells, which were grown to ~25% confluency in 60-mm dishes, were transiently transfected with 2 μg Flag-Podocin and 7 μg pBluescript KS^+^ using the calcium phosphate coprecipitation method (30). Following 12-h incubation, the precipitate was removed by washing twice with 2 ml PBS and the cells were incubated for an additional 36 h in DMEM supplemented with 10% fetal bovine serum at 37°C. Thereafter, the cells were lysed by boiling in Laemmli buffer and the insoluble material was removed by centrifugation (31). The resultant protein lysate was subjected to SDS-PAGE and the separated proteins were transferred to a PVDF membrane (Immobilon; Millipore) and processed for immunoblotting with the indicated antibodies (32).

### MOs and efficacy checking

MOs were designed for the splice blocking of *nephrin*-exon 25 (*nephrinMOex25*: 5′-TGC ACC AAC ACG ACT CAC CTC TGC TC-3′) and of *podocin*-exon 3 (*podocinMOex3*: 5′-TGT AGT CAC TTT TGC AGA CCT GGG CT-3′) (Gene Tools LLC, Philomath, OR, USA). The two morpholinos target the splice acceptor site. The morpholinos were diluted with the injection solution containing 100 mM KCl and 10 mM HEPES (pH 7.6) and were injected at a final concentration of 0.15 mM (*nephrinMOex25*) and 0.25 mM (*podocinMOex3*) into one- or two-cell stage embryos using a Nanoliter 2000 microinjector (World Precision Instruments, Sarasota, FL, USA). The injected volume was ~4.6 nl. Splice blocking was verified using RT-PCR and nested PCR. Total RNA was isolated from five embryos with an RNAqueous-4PCR kit (Life Technologies). RT-PCR was performed using SuperScript III One-Step RT-PCR System with Platinum Taq High Fidelity (Life Technologies) followed by nested PCR using Phusion High-Fidelity DNA Polymerase (New England Biolabs). The primer sets used were: forward, 5′-GGC AGG ATC TGC AAG CTA CAT-3 and 5′-CTC AGG GCC TTC AGG GT GAG-3′ (RT-PCR for *nephrin*); 5′-CTC CTG AAC CCA TTC ATC TGC-3′ and 5′-CTC ATA GAC GCT GCT GTC AGG-3′ (nested PCR for *nephrin*); 5′-GAT GCT TCC TGC GGA GAT AGA-3′ and 5′-TTC CTG TCC AGC AAA ATG TCA-3′ (RT-PCR for *podocin*) and 5′-TCA TCT CTA GCA GCA CGG TTG-3′ and 5′-TCT GGA ATG CTA GCG AAG GAG-3′ (nested PCR for *podocin*). Altered PCR products were subcloned into pCR-BluntII-TOPO and sequenced.

### Hematoxylin and eosin staining

Hematoxylin and eosin staining was conducted as previously described (32). In brief, larvae were fixed with histology fixative (1.5% glutaraldehyde, 4% PFA, 3% sucrose in 0.1 M phosphate buffer, pH 7.3) overnight at 4°C, dehydrated with a graded series of methanol and embedded in JB-4 resin. Four micron sections were sliced and stained with Harris hematoxylin and special eosin II (BBC Biochemical, Mount Vernon, WA, USA). The stained sections were photographed with a Provis AX-70 microscope equipped with a RETIGA EXi digital camera (QImaging).

### Transmission electron microscopy

Transmission electron microscopy was conducted as previously described (3). Larvae were fixed with histology fixative overnight at 4°C. The samples were immersed in 1% OsO_4_ in 0.1 M phosphate buffer for 1 h, dehydrated with a graded series of ethanol and then embedded in Epon-Araldite resin (Electron Microscopy Sciences, Hatfield, PA, USA). Ultrathin silver-gold sections were produced with an ultra 45° diamond knife (Diatome, Biel, Switzerland) and were transferred to copper grids (50 mesh, Nisshin EM) with a carbon-coated Formvar membrane. The sections were stained with uranyl acetate and lead citrate and photographed on a H-7600 transmission electron microscope (Hitachi High Technologies Inc., Schaumburg, IL, USA) equipped with a Kodak 2K × 2K digital camera.

### Co-immunoprecipitation assay

cDNA encoding the intracellular region of zebrafish Nephrin (amino acids 1,065–1,242) was generated by performing PCR on the total RNA from 2 dpf zebrafish larvae as a template, subcloned into pCS3^+^-6Myc vector and verified by DNA sequencing. Generation of Flag-Podocin vector was mentioned earlier. Each 1 μg of indicated Flag-Podocin and 6Myc-Nephrin-C vectors were transfected together with 7 μg pBluescript KS^+^ into 293T cells essentially as previously described (31). Following 36 h incubation, the cells were lysed in 600 μl 50 mM Tris (pH 7.4), 50 mM NaF, 150 mM NaCl, 0.2 mM DTT, 0.5% NP-40, 1 mM PMSF, 10 μg/ml leupeptin, 2 μg/ml aprotinin, 1 μg/ml pepstatin A and 0.2 mM Na_3_VO_4_. Cell lysates were processed as described earlier utilizing the anti-Flag M2 monoclonal antibody (Sigma-Aldrich, St. Louis, MO, USA) for the immunoprecipitation (34). Immunoprecipitated complexes were resolved by SDS-PAGE and co-precipitated proteins were detected by anti-Myc immuno-blot analysis (35).

### Synthesis and egg injection of capped RNA

The human *NEPHRIN* and *PODOCIN* cDNAs were kind gifts from Dr Lawrence Holzman. PCR was used to introduce a non-sense mutation into human *NEPHRIN* cDNA causing R1109X and a single base substitution into human *PODOCIN* cDNA causing R138Q. The following primer sets were used for the generation of mutated cDNA: for *NEPHRIN* mutant: *HNEPHRIN-*forward: 5′-GTC AAA GCT TAT GGC CCT GGG GAC GAC GCT CAG and -reverse: 5′-AAG GAT AGC GGC CGC CTA CAC CAG ATG TCC CCT CAG CTC GAA G; *HNE- PHRIN-1109X*-forward: 5′-GGG TCG GAA GAG GAC TAG GTC AGG AAC GAA TAT GAG GAG AGC C-3′ and -reverse: 5′-GGC TCT CCT CAT ATT CGT TCC TGA CCT AGT CCT CTT CCG ACC CTG CC-3′; for *PODOCIN* mutant: *HPODOCIN-*forward: 5′-GTC AAA GCT TAC CAT GGA GAG GAG GGC GCG GAG CTC CTC CAG-3′ and -reverse: 5′-AAG GAT AGC GGC CGC CTA TAA CAT GGG AGA GTC TTT CTT TTT AGG-3′, *HPODOCIN-138Q-*forward: 5′-AGA GTA ATT ATA TTC CAG CTG GGA CAT CTG CTT CCT GGA AGA G-3′ and -reverse: 5′-CAG ATG TCC CAG CTG GAA TAT AAT TAC TCT TTC ATA CTC TTG TAC AAC C-3′. The PCR products were subcloned into pCR-BluntII-TOPO and verified by DNA sequencing. These vectors were digested with restriction enzymes and used as templates for the synthesis of capped RNA. Capped RNA was synthesized using the mMESSAGE mMACHINE kit (Life Technologies). Synthesized capped RNA (~0.64 pg for human wild-type and mutated *NEPHRIN* and ~32 pg for human wild-type and mutated *PODOCIN*) was injected into one-cell stage embryos along with morpholinos (0.15 mM *nephrinMOex25* or 0.25 mM *podocinMOex3*).

## Results

### mRNA and protein expression

**Figure 1:**
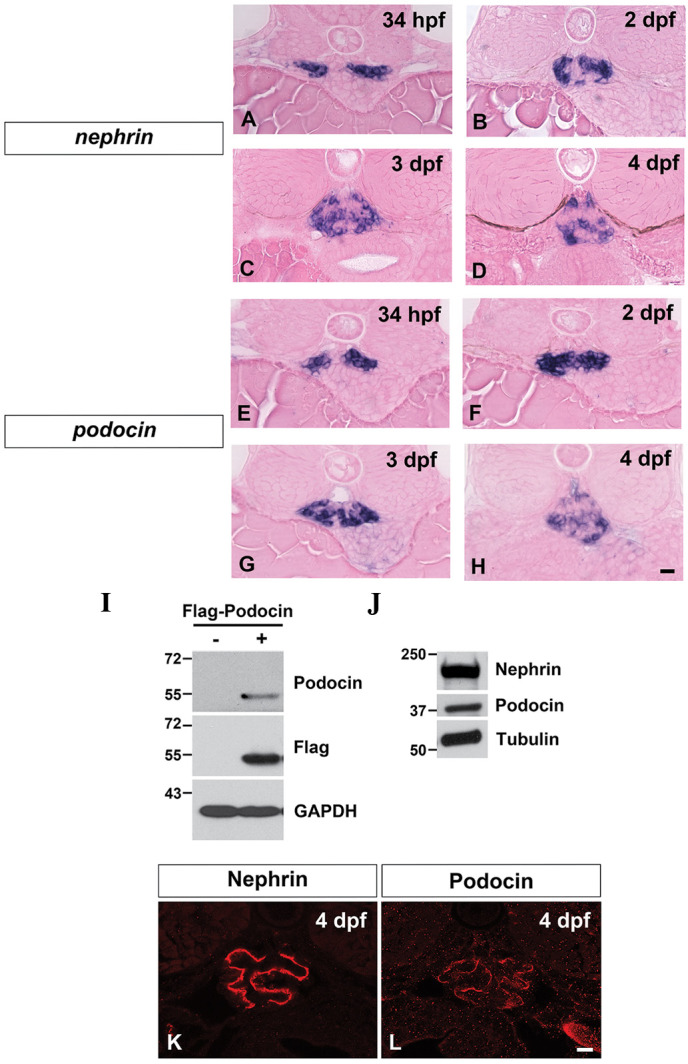
mRNA and protein expression. (A–H) mRNA expression of (A–D) *nephrin* and (E–H) *podocin* is already detected in the zebrafish pronephric glomerular primordial at 34 hpf and sustained throughout the following developmental period (2–4 dpf). (I) Specificity of newly-developed anti-zebrafish Podocin antibody. The antibody detects a Flag-tagged full-length zebrafish Podocin. (J) Intrinsically expressed Nephrin and Podocin in 4 dpf zebrafish are detected by immunoblot analysis using the anti-Nephrin and anti-Podocin antibodies. (K and L) Nephrin and Podocin proteins were detected as a pair of tortuous line segments by immunohistochemistry. (H and L) Bar scales, 10 μm. dpf, days post-fertilization; hpf, hours post-fertilization.

Throughout zebrafish pronephric development [34 h post-fertilization (hpf) to 4 dpf], *nephrin* and *podocin* mRNA expression was detected in the glomerular primordia and compared with the glomerulus (Fig. 1A–H). The expression patterns of *nephrin* and *podocin* markedly resembled that for Wilms’ tumor 1a, which is predominantly expressed in the podocytes within the pronephric glomerulus (1).

Antibodies specific to Nephrin and to Podocin were developed to determine their expression pattern and localization. The specificity of the anti-Nephrin antibody was previously described (3). To examine the specificity of the anti-Podocin antibody, the possibility of a reaction with Flag-tagged zebrafish Podocin expressed in 293T cells was investigated. Immunoblot analysis showed that the anti-Podocin antibody was capable of detecting an ~55 kDa protein, corresponding to the Flag-tagged Podocin (Fig. 1I). These anti-Nephrin and anti-Podocin antibodies could also detect ~240 and ~40 kDa protein bands corresponding to endogenous Nephrin and Podocin, respectively (Fig. 1J).

With these antibodies, Nephrin and Podocin were immunohistochemically detected in 4 dpf larvae as two continuous tortuous lines, which were likely to be situated along the glomerular basement membrane (Fig. 1K and L). A similar tortuous linear pattern for Nephrin localization was also observed for the rodent metanephric glomerulus (36,37).

### Splice blocking of nephrin and podocin pre-mRNA

**Figure 2:**
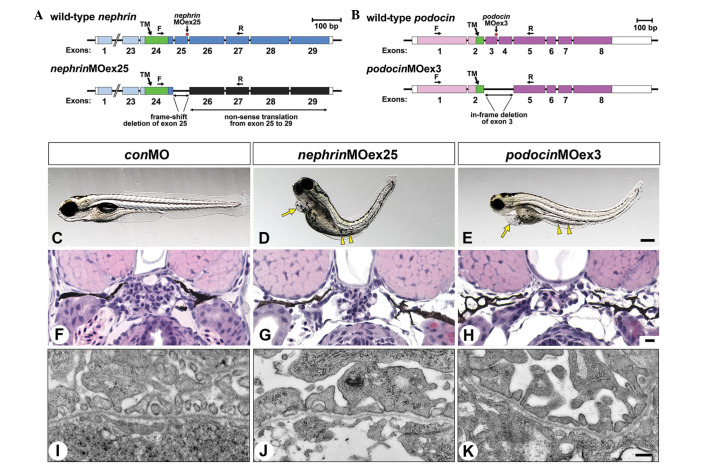
Gene knockdown using splice-blocking morpholinos. (A) *nephrinMOex25* targets the splice-acceptor site of *nephrin* exon 25 and induces a frame-shift deletion. (B) *PodocinMOex3* targets the splice-acceptor site of podocin exon 3 and induces an in-frame deletion of exons 3 and 4. (F) and (R) show the sites of primer sets for the testing of the efficacy of target modification (PCR data shown in Fig. 1A and B). (C–E) Lateral view of 4 dpf larvae. *NephrinMOex25* and *podocinMOex3* morphants exhibit pericardial edema (arrow), edematous swelling of yolk sac extension (arrowheads) and body-axis curvatures. (F–H) Glomerulus in the 4 dpf larvae in HE-stained sections. The control larva exhibits a well-developed glomerular capillary and mesangium. In the morphants, glomerular development is markedly disrupted. (I–K) Ultrastructure of glomerular capillary wall. The control larva exhibits fine regular foot processes and slit diaphragm, present in a ‘beads on a string’ pattern. In the *nephrinMOex25* morphant, the regular foot processes are not observed at all, however, in the *podocinMOex3* morphant, foot processes with slit diaphragm are formed. TM, transmembrane domain coding region. Bar scales, 250 μm in (E); 10 μm in (H) and 50 nm in (K). dpf, days post-fertilization.

To determine the function of Nephrin and Podocin in the development of the zebrafish pronephric glomerulus, splice blocking MOs were designed for Nephrin and Podocin to target the splice acceptor sites of exon 25 for *nephrin* (*nephrinMOex25*) and exon 3 for *podocin* (*podocinMOex3*), respectively (Fig. 2A and B).

**Figure 3:**
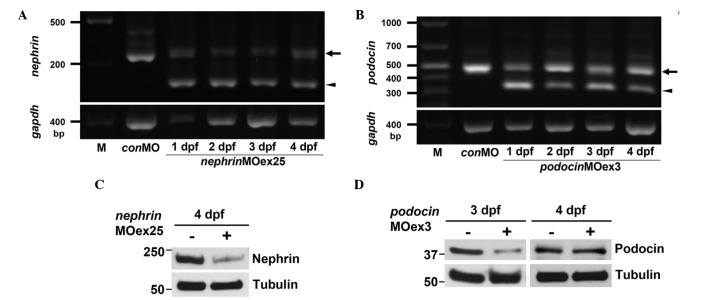
Morpholino efficacy. (A and B) Injection of *nephrinMOex25* and *podocinMOex3* resulted in the mis-splicing of mRNA as detected by an altered RT-PCR product (arrowheads). Arrows indicate intact mRNA. (A) The efficacy for *nephrinMOex25* is consistent from 1 to 4 dpf. (B) By contrast, the efficacy for *podocinMOex3* is recognized from 1 to 4 dpf, although a significant expression of intact podocin mRNA is also detected. (C) In the *nephrinMOex25* morphants, the protein expression of Nephrin was reduced at 4 dpf larvae. (D) In the *podocinMOex3* morphants, a reduced expression of Podocin protein is observed at 3 dpf, however, the expression is recovered at the same level of control at 4 dpf. dpf, days post-fertilization.

Injection of *nephrinMOex25* resulted in mis-spliced mRNA as detected by an altered RT-PCR product (Fig. 3A). This altered product was ~50 bp smaller than the wild-type. Sequencing of the altered RT-PCR product revealed a deletion of exon 25 that resulted in a frame shift and non-sense translation of the subsequent exons, resulting in an almost complete loss of the intracellular domain of the Nephrin protein in *nephrinMOex25* morphants (Fig. 2A). The efficacy for *nephrinMOex25* was consistently recognized from 1 to 4 dpf (Fig. 3A) and the protein expression of Nephrin was reduced at 4 dpf (Fig. 3C).

Administration of *podocinMOex3* also resulted in mis-spliced mRNA as detected by an altered RT-PCR product that was ~150 bp smaller than the wild-type amplicon (Fig. 3B). Sequencing of the altered RT-PCR product revealed an in-frame deletion of exons 3 and 4 (Fig. 2B). The truncated RT-PCR product was recognized from 1 to 4 dpf, although a significant expression of intact Podocin mRNA was also detected (Fig. 3B). The reduced expression of the Podocin protein was observed in the 3 dpf morphants, however, the expression was recovered at the same level as in the control at 4 dpf (Fig. 3D).

*NephrinMOex25* and *PodocinMOex3* morphants exhibited pericardial and yolk edema from 3 to 4 dpf (Fig. 2C–E). In addition to edema, the majority of the morphants showed body-axis curvature (Fig. 2C–E). In wild-type zebrafish, a pair of pronephric glomerular primordia merged with glomerular capillaries and mesangium at the midline to form a single glomerulus (Fig. 2F) (1). The glomerulus was smaller in size in the morphants compared with that in the wild-type larvae and contained poorly developed glomerular capillaries and mesangium (Fig. 2G and H).

In 4 dpf control larvae, regular foot processes with SD covered a large area of the urinary surface of the glomerular basement membrane (Fig. 2I). At the same stage, *nephrinMOex25* morphants were associated with a great reduction in the area covered with regular foot processes. Instead of the foot processes, irregularly shaped processes covered the larger area of its urinary surface and the SD was not observed between these irregularly shaped processes (Fig. 2J). In 4 dpf *podocinMOex3* morphants, the foot processes with SD covered a large area of the glomerular wall, however, the processes varied in shape and size (Fig. 2K).

### Rescue of the morphant phenotypes by human intact mRNA

**Figure 4:**
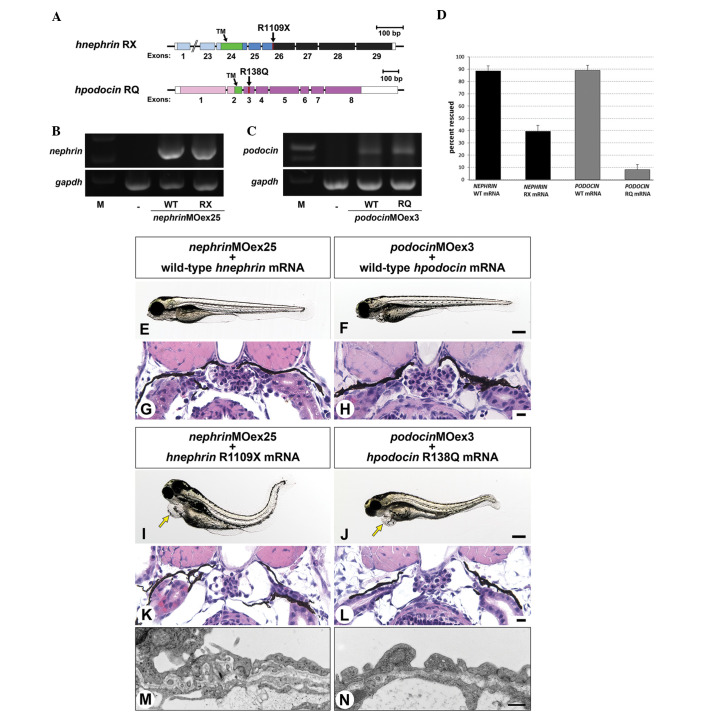
Human mRNA injection. (A) The mutation sites introduced in the human Nephrin and Podocin mRNAs. (B and C) Three types of injected human mRNA (wild-type, *NEPHRIN-R1109X* and *PODOCIN-R138Q*) are detected in 2 dpf larvae by RT-PCR. (D) Wild-type mRNAs of human Nephrin and Podocin may rescue ~89% of *nephrinMOex25* and *podocinMOex3* morphants, respectively, however, the percentage of rescued larvae is significantly reduced when mutated mRNA is co-injected at 39 and 8.1%, respectively. The phenotype was scored by pericardial edema, body-axis curvature, glomerulus morphology and altered glomerular barrier. (E–H) Wild-type human Nephrin and Podocin mRNA rescue the morphant phenotypes. (E and F) The larvae exhibit a straight body-axis with no or slight pericardial edema, (G and H) and a well-developed glomerulus. (I–N) Mutated human *NEPHRIN-R1109X* and *PODOCIN-R138Q* mRNA do not rescue the morphant phenotypes as frequently. (I and J) Larvae injected with mutated mRNA exhibit pericardial edema and body-axis curvature, (K and L) hypoplastic glomerulus (M and N) and no regular foot processes. Bar scales, 250 μm in (F), (J); 10 μm in (H), (L) and 100 nm in (N). dpf, days post-fertilization.

To investigate whether administration of human intact mRNA rescues the morphant phenotypes, splice blocking morpholino and human wild-type mRNA were simultaneously injected into one-cell stage embryos. Human *NEPHRIN* and *PODOCIN* mRNA were detected in the 4 dpf-injected larvae by RT-PCR (Fig. 4B), indicating that the injected mRNA remained until at least four days following injection. *nephrinMOex25* and *podocinMOex3* morpholino-injected embryos exhibited pericardial edema, yolk edema and body-axis curvature. Moreover, the glomerulus was smaller in size compared with the wild-type larvae and contained poorly developed glomerular capillaries and mesangium (Fig. 2F–H). All these features may be rescued by ~89% by co-injection of human Nephrin and Podocin mRNAs (Fig. 4D). In addition, rescued morphants exhibited a straight body axis (Fig. 4E and F) and a well-developed pronephric glomerulus at 4 dpf (Fig. 4G and H), although slight pericardial edema was observed in the larvae injected with human *PODOCIN* mRNA and *podocinMOex3* (Fig. 4F). These data therefore indicate that the functions of human and zebrafish Nephrin and Podocin are interchangeable.

### The phenotype induced by a nephrinMOex25 or podocinMOex3 in zebrafish embryos may be rescued by wild-type human NEPHRIN or PODOCIN but not by NEPHRIN-R1109X and PODOCIN-R138Q mutant mRNA

A number of types of disease-causing mutations in Nephrin and Podocin proteins have been reported in human patients, including R1109X in Nephrin (25) and R138Q in Podocin (26). NEPHRIN-R1109X is a non-sense mutation leading to a large deletion of the intracellular domain of Nephrin and it is one of the two major disease-causing mutations in Finnish-type congenital nephrotic syndrome (24) (Fig. 4A). PODOCIN-R138Q is a missense mutation resulting in the substitution of arginine at position 138 for the uncharged amino acid glutamine and is also one of the most common disease-causing mutations in steroid-resistant nephrotic syndrome (23) (Fig. 4A).

To determine the effect of mutated mRNA on the rescue of the morphant phenotypes, *nephrinMOex25* and *podocinMOex3* were injected together with mutated mRNA encoding human *NEPHRIN-R1109X* and *PODOCIN-R138Q*, respectively, into one-cell stage embryos. The persistence of the two mutated mRNAs was detected in 4 dpf larvae by RT-PCR (Fig. 4B and C), but the ratio of rescued larvae was significantly lower for the mutated mRNAs compared with the wild-type mRNA (Fig. 4D). Non-rescued larvae exhibited pericardial edema, body-axis curvature and hypoplastic glomerulus, as observed in the larvae injected with the morpholino only (Fig. 4G, H, K and L). The formation of regular foot processes was affected in the larvae injected with *NEPHRIN-R1109X* and *nephrinMOex25,* as with *nephrinMOex25* alone (Fig. 4M). Notably, the larvae injected with *PODOCIN-R138Q* and *podocinMOex3* did not exhibit any regular foot processes with SD (Fig. 4N). However, larvae injected with *podocinMOex3* formed foot processes to some extent (Fig. 2K), indicating that *PODOCIN-R138Q* exacerbated the morphant phenotype observed with *podcinMOex3* alone. The phenotype was scored by morphology and altered glomerular barrier and, in contrast to human Nephrin and Podocin mRNAs, co-injection of and *nephrinMOex25* and mutant *NEPHRIN-R1109X* or *podocinMOex3* and *PODOCIN-R138Q* only rescued 39 and 8.1%, respectively.

### Protein interaction between Nephrin and Podocin

**Figure 5:**
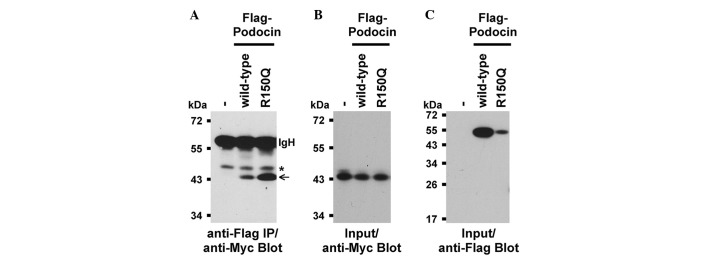
(A) Co-immunoprecipitation assay for Myc-Nephrin and Flag-Podocin. Following anti-Flag IP, coprecipitated Nephrin was revealed by anti-Myc immuno-blotting (see arrow). Asterisk marks a non-specific protein band. IgH, immunoglobulin heavy chain. (B) Similar input levels of Myc-Nephrin. (C) Expression level of wild-type and Flag-tagged Podocin is notably higher in comparison with that of the R150Q mutant. Myc-Nephrin, Myc-tagged zebrafish Nephrin intracelluar region; Flag-Podocin, Flag-tagged zebrafish full-length Podocin; IP, immunoprecipitation.

To examine whether the protein properties of a mutated PODOCIN were biochemically altered, the abilities of Flag-tagged wild-type and mutated (R150Q) zebrafish full-length Podocin to bind to the Myc-tagged C-terminal intracellular domain of zebrafish Nephrin were compared. Notably, the R150Q zebrafish Podocin mutation corresponded to the disease-causing R138Q mutation in human Podocin. Consistent with previous studies for mammalian Nephrin and Podocin (17,38), binding between wild-type zebrafish Podocin and the intracellular region of Nephrin (Fig. 5) was observed. Binding between the mutated Podocin-R150Q and Nephrin (Fig. 5) was also detected. However, mutated Podocin interacted significantly more with Nephrin despite the lower expression levels of mutated Podocin compared with wild-type Podocin (Fig. 5). The latter may suggest that the mutation of Podocin makes it more prone to protein degradation.

## Discussion

In the mammalian kidney, Nephrin and Podocin localize at the SD between the podocyte foot processes and play essential roles in the formation and maintenance of the SD. A previous study of the localization of zebrafish Nephrin during development revealed its predominant localization at the podocytes within the pronephros, which is similar to its localization in the mammalian metanephros (3). Similarly, Podocin was previously identified to localize in two tortuous line segments within the pronephros, which was similar to that of Nephrin, indicating that Podocin also predominantly localizes at the podocytes in the zebrafish pronephros.

*NephrinMOex25* and *PodocinMOex3* induced the altered splicing of pre-mRNA to greatly attenuate the expression of full-length protein products. Nephrin and Podocin were already expressed in the primitive podocytes of glomerular primordia, which had not yet formed foot processes and SD, suggesting that Nephrin and Podocin contribute to the differentiation of primitive podocytes. The attenuated expression of these two proteins is thus likely to affect the appropriate development and maturation of podocytes. In zebrafish and mammals, podocytes are known to produce vascular endothelial growth factor, which contributes to the migration of primitive capillary endothelial cells and the maturation of glomerular capillaries (27,39). Therefore, impairment of podocyte differentiation may subsequently affect the formation and maturation of other glomerular structures (so-called endocapillary region). The aforementioned scenario may explain the underlying mechanism for the glomerular hypoplasia in the morphants examined.

Although identity in amino acid sequence is low in Nephrin and Podocin homologues between zebrafish and humans, the alignment of the functional domains within the proteins is highly conserved (9,21). Notably, human wild-type Nephrin and Podocin mRNAs may rescue the phenotype of the *nephrinMOex25* and *podocinMOex3* morphants, respectively. Thus, the current data suggest that the protein function of Nephrin and Podocin is highly conserved between zebrafish and humans.

Human Nephrin and Podocin mRNAs containing disease-causative mutations exhibited significantly lower efficiency in rescuing the morphant phenotypes than the wild-type mRNAs. Moreover, the mRNA coding *PODOCIN-R138Q* mutant prominently affected the formation of regular foot processes interspaced with SD, although the injection of *podocinMOex3* alone did not largely disturb the SD formation. These results suggest that the morpholino and mutated mRNA synergistically interfered with the formation of the foot processes interspaced with SD. Therefore, this experimental system offers potential for investigation of the spatiotemporal consequences of gene mutations associated with the nephrotic syndrome in the early phase of glomerular development. In addition, it may be useful to explore the pathogenic mechanisms underlying human congenital nephrotic syndromes.

Multi-protein complexes, including those containing Nephrin and Podocin are formed at the SD and are believed to be crucial in the establishment and maintenance of the SD. The current study demonstrates that an interaction occurred between wild-type Nephrin and Podocin in zebrafish. Nishibori *et al* (40) reported that the human *PODOCIN-R138Q* mutant, which corresponds to the zebrafish *podocin*-R150Q, is trapped in the endoplasmic reticulum and the trapped Podocin is hypothesized to interfere with trafficking of Nephrin (40). The present pull-down assay indicated that the binding activity of zebrafish *podocin*-R150Q to Nephrin was stronger compared with wild-type Podocin. If the zebrafish Podocin-R150Q is also trapped and accumulated in the endoplasmic reticulum, the mutated Podocin most likely impaired Nephrin trafficking from the endoplasmic reticulum to its proper destination in the SD near the foot processes due to its abnormally strong ability to bind Nephrin. This hypothesis is in agreement with the observation that the formation of regular foot processes and SD was more detrimentally affected in the larvae injected with human *PODOCIN-R138Q* mRNA plus *podocinMOex3* than with the morpholino alone.

The present study highlights the high evolutionary conservation in the function of Podocin and Nephrin from zebrafish to humans and establishes a system by which disease-causing mutations in human *PODOCIN* and *NEPHRIN* may be assessed for their biological consequences. This information is likely to facilitate studies to gain an improved understanding of the development of several kidney diseases, including nephrotic syndrome.
